# A Comparison of QM/MM Simulations with and without the Drude Oscillator Model Based on Hydration Free Energies of Simple Solutes

**DOI:** 10.3390/molecules23102695

**Published:** 2018-10-19

**Authors:** Gerhard König, Frank C. Pickard, Jing Huang, Walter Thiel, Alexander D. MacKerell, Bernard R. Brooks, Darrin M. York

**Affiliations:** 1 Laboratory of Computational Biology, National Heart, Lung, and Blood Institute, National Institutes of Health, Bethesda, MD 20892, USA; pickard81@gmail.com (F.C.P.IV); jing.huang.ff@gmail.com (J.H.); brb@nhlbi.nih.gov (B.R.B.); 2 Laboratory for Biomolecular Simulation Research, Institute for Quantitative Biomedicine, Department of Chemistry and Chemical Biology, Rutgers University, Piscataway, NJ 08854, USA; 3 Max-Planck-Institut für Kohlenforschung, 45470 Mülheim an der Ruhr, Germany; thiel@mpi-muelheim.mpg.de; 4 Department of Pharmaceutical Science, School of Pharmacy, University of Maryland, 20 Penn Street, Baltimore, MD 21201, USA; alex@outerbanks.umaryland.edu; 5 School of Life Sciences, Westlake University, 18 Shilongshan Street, Hangzhou 310024, China

**Keywords:** hydration free energy, QM/MM, polarization

## Abstract

Maintaining a proper balance between specific intermolecular interactions and non-specific solvent interactions is of critical importance in molecular simulations, especially when predicting binding affinities or reaction rates in the condensed phase. The most rigorous metric for characterizing solvent affinity are solvation free energies, which correspond to a transfer from the gas phase into solution. Due to the drastic change of the electrostatic environment during this process, it is also a stringent test of polarization response in the model. Here, we employ both the CHARMM fixed charge and polarizable force fields to predict hydration free energies of twelve simple solutes. The resulting classical ensembles are then reweighted to obtain QM/MM hydration free energies using a variety of QM methods, including MP2, Hartree–Fock, density functional methods (BLYP, B3LYP, M06-2X) and semi-empirical methods (OM2 and AM1 ). Our simulations test the compatibility of quantum-mechanical methods with molecular-mechanical water models and solute Lennard–Jones parameters. In all cases, the resulting QM/MM hydration free energies were inferior to purely classical results, with the QM/MM Drude force field predictions being only marginally better than the QM/MM fixed charge results. In addition, the QM/MM results for different quantum methods are highly divergent, with almost inverted trends for polarizable and fixed charge water models. While this does not necessarily imply deficiencies in the QM models themselves, it underscores the need to develop consistent and balanced QM/MM interactions. Both the QM and the MM component of a QM/MM simulation have to match, in order to avoid artifacts due to biased solute–solvent interactions. Finally, we discuss strategies to improve the convergence and efficiency of multi-scale free energy simulations by automatically adapting the molecular-mechanics force field to the target quantum method.

## 1. Introduction

Biological systems are mostly composed of water, and the interactions with water are a central feature of life as we know it [1,2,3,4,5]. Solvation influences a wide variety of processes, including protein folding [6,7,8,9,10], crystal polymorphism [11], conformational equilibria [12,13,14,15] and even basic reaction pathways [16]. Furthermore, water is one of the main actors in the selectivity of biochemical interactions and has a profound influence on both the kinetics and thermodynamics of protein-protein, protein-nucleic acid and protein-ligand binding [17]. Any binding event between a ligand and a receptor in aqueous solution is first preceded by the desolvation of water molecules from the binding site and the ligand’s surface. A binding event only occurs if the ligand-receptor interactions can compensate for the loss of ligand-solvent and receptor-solvent interactions and the associated entropy changes [18,19,20]. Given the fundamental importance of the solvent, no biomolecular model is adequate without properly accounting for solvation.

The free energy costs of (de-)solvation are quantified by its solvation free energy, which corresponds to the transfer free energy of the molecule from the gas phase to solution [21,22,23,24,25]. In aqueous solution, the solvation free energy is also known as hydration free energy ($\Delta G_{\mathrm{hyd}}$). In the molecular mechanics (MM) modeling community, $\Delta G_{\mathrm{hyd}}$ values have been an essential benchmark quantity for decades [14,26,27,28,29,30,31,32,33,34,35,36,37,38,39,40,41,42,43,44,45,46,47,48,49,50,51,52,53,54]. Furthermore, significant efforts have been invested in the quantum mechanical (QM) community to develop highly accurate implicit solvent methods [55,56,57,58,59,60,61,62,63]. However, when it comes to a hybrid QM/MM approach, where a quantum mechanical solute is embedded in a classical explicit solvent, solvation free energies have received less attention because of the computational cost and complexity of sampling the solvent degrees of freedom.

Gao was a pioneer in determining QM/MM solute–solvent interaction energies for amino acid side chain analogs and nucleotide bases [64], as well as absolute solvation free energies [65]. This work was a milestone for QM/MM, and significant efforts have since been invested by many groups all around the world [66,67,68,69,70,71,72,73,74,75,76,77,78,79,80], making it an indispensable tool in computational chemistry [81,82,83]. It is therefore also not surprising that QM/MM techniques have recently received increasing attention in the context of free energy calculations [84,85,86,87,88,89,90,91,92,93,94,95,96,97,98,99,100,101,102,103,104,105]. Focusing on solvation, Stanton, Hartsough and Merz used QM/MM to determine the solvation free energies of ions [106]. Shoeib et al. studied absolute hydration free energies of ions and small solutes [107]. Using the quasichemical theory of solutions, Asthagiri, Pratt and Kress calculated the hydration free energy of PBE water [108], and Weber and Asthagiri provided the hydration free energy of BLYP-D water [109]. Vapor-liquid equilibria of QM water were studied by McGrath et al. [110]. Radial distribution functions of QM water have received the attention of multiple groups [110,111,112,113,114,115,116,117,118], as well as the interaction energies of multimers [119,120,121,122,123]. Relative solvation free energies were calculated by Reddy, Singh and Erion [124,125,126]. Kamerlin, Haranczyk and Warshel discussed solvation free energies of acetate and methylamine in the context of $pK_{a}$ calculations [127]. Shields, Temelso and Archer determined binding free energies of water to small water clusters [128,129]. More recently, we have applied QM/MM solvation free energy calculations within the framework of the SAMPL challenges [79,130,131].

One of the most important shortcomings of conventional force fields is the neglect of electronic polarization. During a simulation, the charge distribution of an MM molecule cannot respond to its environment. Since polarizability is known to be important, especially in QM/MM simulations, there is major interest in the use of polarizable force fields such as the CHARMM Drude force field [132]. Here, we perform simulations with both the CHARMM fixed charge force field and the CHARMM Drude polarizable force field, to discern the benefits and challenges of this new generation of force fields and help lay the groundwork for future development of QM/MM methods with increased predictive capability. It is of particular practical interest to ascertain the degree to which optimization of the QM/MM van der Waals interaction parameters may be needed for different QM methods, and the additional computational efforts of the Drude force field are beneficial. Our recent work [133] analytically showed that significant additional computational costs can be justified in multi-scale free energy simulations, if the sampling method exhibits a higher phase space overlap with the target QM Hamiltonian. Thus, it can be expected that polarizable force fields and, ultimately, quantum-mechanical methods will play an increasing role in free energy calculations [134,135,136,137,138,139].

The remainder of this paper is organized as follows: First, we summarize the details of the model systems and simulations. Next, we present the results for the $\Delta G_{\mathrm{hyd}}$ values of twelve simple solutes, using both the fixed charge and the Drude force field. Finally, we compare the performance of MP2, Hartree–Fock, several density functional methods (BLYP, B3LYP, M06-2X) and semi-empirical methods (OM2 and AM1 ) in terms of $\Delta G_{\mathrm{hyd}}$ with QM/MM. This is done for both the fixed charge force field and the Drude force field. We also discuss other aspects that can have an impact on the accuracy of the results and the efficiency of the free energy simulations, including empirically scaling $\Delta G_{\mathrm{hyd}}$ values, using a self-consistent optimization of the Drude particles at each step, or increasing the overlap between the MM force field and the QM target energy function by introducing a tailored MM’ force field. The Appendix includes a comparison of the convergence properties of free energy estimates based on the fixed charge and the Drude force field and also provides the detailed results of all MM free energy sub-steps.

## 2. Methods

### 2.1. Simulations

**Figure 1:**
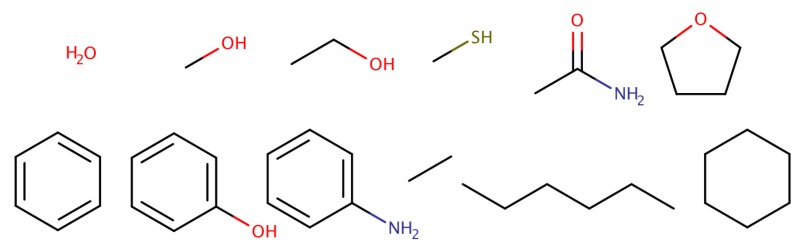
Twelve simple molecules were employed for the determination of hydration free energies.

A test set of 12 molecules was used: water, methanol, ethanol, methanethiol, acetamide, tetrahydrofuran, benzene, phenol, aniline, ethane, *n*-hexane and cyclohexane (see Figure 1). These molecules were chosen to cover a large range of hydration free energy values, between −8.05 kcal/mol (acetamide) [140] and +2.55 kcal/mol (*n*-hexane) [24], encompassing different levels of hydrophobicity. In addition, this set includes amino acid side-chain analogs, ring compounds and hydrophobic molecules, thus providing a minimalistic test set without additional challenges, such as protonation, tautomerism or extensive conformational flexibility. We have previously used this test set to study polarization energies [141], the convergence of free energy simulations [133] and the use of 1-butanol for the extraction of polar solutes [142].

All free energy calculations were conducted using the CHARMM software [143,144], with the CHARMM General Force Field (CGenFF) for organic molecules [145] (the fixed charge force field) and the CHARMM Drude force field (polarizable force field) [146,147,148,149,150,151]. To determine $\Delta G_{\mathrm{hyd}}$, each molecule was alchemically annihilated both in the aqueous phase and in the gas phase. The gas phase simulations used Langevin dynamics with a friction coefficient of 1 ps${}^{-1}$ and a temperature of 300 K. The simulation time was 500 ns, and coordinates were saved every 20 ps.

We modeled the aqueous phase with 1687 water molecules in a cubic simulation cell with edge lengths between 36.85 and 36.89 Å, as determined from equilibration simulations of 0.5 ns in the isobaric isothermal ensemble (NPT). For the fixed charge simulations, we used CHARMM TIP3P water with Lennard–Jones parameters on the hydrogens [152] and a Nosé–Hoover thermostat [153,154] at 300 K. We performed the Drude simulations with the SWM4 water model [155] and an operator-splitting velocity-Verlet algorithm [156], using a response time τ of 0.1 and a temperature of 300 K for the atomic particles and a τ of 0.005 and a temperature of 1 K for the relative motion of Drude particles. The friction constant was set to 10 ps${}^{-1}$. In all solvent simulations, long-range electrostatic interactions were computed with the particle Mesh Ewald method [157], and Lennard–Jones interactions were switched off between 10 and 12 Å. All molecules were first equilibrated for 0.5 ns using constant pressure, followed by an equilibration of each alchemical transformation state (λ state) for 0.5 ns in the constant volume ensemble (NVT). Production simulations in aqueous solution were conducted for 5 ns. All simulations used a time step of 1 fs, saving frames every 1000 steps. SHAKE [158] was used to keep all bonds in the solvent rigid.

Both the simulations in the aqueous phase and in the gas phase employed λ-Hamiltonian replica exchange [159] to enhance sampling by exchanging structures between adjacent λ states every 20,000 steps, using the REPD module of CHARMM. The free energy calculation was broken into two parts. First, the charges were scaled to zero ($\Delta G_{\mathrm{char}}$), using three steps for the fixed charge force field (λ = 0.00, 0.20, 0.55 and 1.00) and five steps for the Drude force field (λ = 0.00, 0.10, 0.25, 0.5, 0.75 and 1.00). The choice of this protocol is discussed in more detail in Appendix A. Second, the van der Waals interactions were turned off ($\Delta G_{\mathrm{vdw}}$) with λ = 0.00, 0.15, 0.30, 0.45, 0.60, 0.75, 0.87, 0.96 and 1.00 for the fixed charge force field and λ = 0.0, 0.1, 0.2, 0.3, 0.4, 0.5, 0.6, 0.7, 0.8, 0.9 and 1.0 for the Drude force field. Soft core potentials, as implemented with the PSSP command in the PERT module of CHARMM, were used with the default parameters to avoid the “van der Waals endpoint problem [143,160].” Based on the information in the Hamiltonian replica exchange log file, free energy differences were calculated with Bennett’s acceptance ratio (BAR) method [161], as implemented in the FREN module of CHARMM. Each free energy simulation was repeated four times to calculate averages and standard deviations.

### 2.2. QM/MM Corrections

The last 9000 frames of the physical end points were employed for multi-scale free energy simulations between the MM and QM Hamiltonians. QM calculations were performed with Q-Chem [162] based on the Q-Chem/CHARMM interface [163]. In particular, potential energy differences were evaluated with four different methods using the 6-31G(d) basis set [164]: BLYP [165,166], B3LYP [167], Hartree–Fock and M06-2X [168,169]. The second-order Møller–Plesset (MP2) [170] results were calculated with the aug-cc-pVDZ basis set [171]. The SCF convergence criterion was set to $10^{-10}$. The semi-empirical QM calculations (SQM) were performed with the MNDO program [172], using OM2 [173,174] and AM1 [175]. Additional calculations were also performed with PM3 [176], but the results were poor (RMSD > 3.5 kcal/mol), in agreement with our previous experiences in the SAMPL5 challenge [130].

Electrostatic embedding was used (i.e., the QM or SQM solute is polarized by the MM point charges of the solvent, but the solute–solvent van der Waals interactions are still calculated on the MM level). Since Q-Chem and MNDO do not support periodic boundary conditions (PBC) in a fully consistent manner, potential energy differences for the free energy corrections were calculated by centering the solvent box around the solute molecule with the MERGE command of CHARMM, followed by a potential energy evaluation of the simulation box without any cutoffs. Only the solute–solute and electrostatic solute–solvent interactions were considered for the potential energy differences, by using the BLOCK module of CHARMM. This is justified by the fact that the solvent–solvent interactions and the solute–solvent van der Waals interactions cancel when calculating the potential energy difference between MM and QM/MM. This way, also long-range electrostatic interactions are still considered, but only at the MM level. This treatment is analogous to that done in [79,130,177]. For the Hartree–Fock QM/MM corrections based on the Drude simulations, we also considered potential energy evaluations where all Drude particles within 6 Å of the solute were first relaxed with 100 steps of conjugate gradient energy minimization using the MM force field, followed by five steps of energy minimization with QM/MM. This allows some response to the polarization from the QM region. All other particles were held in place during the minimization with the CONS FIX command.

The MM→(S)QM/MM free energy corrections were computed with the Zwanzig equation (also known as free energy perturbation or the exponential formula), as implemented in the FREN module of CHARMM [178]. This choice is justified, since the limiting factor for convergence is the phase space overlap between MM and QM [177,179]. A more thorough discussion of this aspect can be found in [133], where Figure 10 is based on exactly the same data as some of the gas phase results here.

### 2.3. Generation of the MM’ Tailored Force Field

To illustrate the effect of adjusting the bonded parameters of the force field on the convergence of multi-scale free energy simulations, tailored force field parameters were generated for each molecule based on OM2. For this purpose, the initial conformation in the gas phase of each molecule was energy minimized. The resulting OM2-optimized structure was then used to populate the bond and angle parameters in CHARMM. To allow unique equilibrium bond length and angle values for all atoms, every atom was assigned to its own unique type. Charge and Lennard–Jones parameters for each unique atom type were obtained from the original parameterization. The overall procedure of generating tailored bonded parameters was implemented as the new QMFIX command in the FREN module of CHARMM. Tailored parameter and topology files were written with the MKDUMMY command in the FREN module.

Based on the original parameters and the tailored MM’ force field, simulations of only the physical end points in both the gas phase and in solution were performed. The simulation length was adjusted to 100 ns in the gas phase and 10 ns in the aqueous phase, with 10,000 frames saved for later analysis and all other settings unchanged. Three different ways to calculate the QM/MM free energy corrections were employed: (a) using the Zwanzig equation based on a simulation with the original force field; (b) using the Zwanzig equation based on a simulation with the MM’ tailored force field; (c) combining the data of the original force field and the MM’ tailored force field simulation with the Non-Boltzmann–Bennett (NBB) method [79,80,180].

## 3. Results and Discussion

Before discussing the impact of using QM/MM on the affinity for water, it is illustrative to observe the faithfulness of the solute–water interactions in pure MM. Hydration free energies have been classical benchmark systems for decades. In the CHARMM force field, the compatibility with a particular water model such as TIP3P is a centerpiece of the parameterization strategy, in particular for the charges. Thus, it is expected that the interactions with water are comparable to experiment.

Table 1 lists the hydration free energies for both the CHARMM fixed charge force field ($\Delta G_{\mathrm{hyd}}^{\mathrm{FC}}$) and the Drude force field ($\Delta G_{\mathrm{hyd}}^{\mathrm{Drude}}$). More detailed results, listing the free energy results of the gas phase, electrostatic and van der Waals changes can be found in Appendix B. Since each simulation was repeated four times, also the corresponding standard deviations of the results are provided. The overall metrics for agreement with experiment are listed in the last three rows. While the fixed charged force field exhibits a root mean squared deviation (RMSD) of 0.89 kcal/mol, the Drude force field reaches an RMSD of 0.55 kcal/mol. Thus, the Drude force field outperforms the fixed charge force field. Both force fields yield what is considered “chemical accuracy”, but this is most likely a reflection of the simplicity of the test set and the high level of optimization of the parameters. In terms of mean signed deviation, the Drude force field also yields a more favorable result (0.04 kcal/mol compared to 0.65 kcal/mol). This indicates some small systematic bias of the fixed charge force field in terms of being overly hydrophobic. The correlation coefficients with the experiment are in both cases excellent (R${}^{2}$ of 0.97 and 0.99).

The last column of Table 1 lists the differences between the fixed charge and the Drude force field results. While the deviations for most apolar molecules are not statistically significant, the results for water, acetamide and phenol differ by more than one kcal/mol. Furthermore, several other polar molecules exhibit a change of their $\Delta G_{\mathrm{hyd}}$, but all changes improve the agreement with experiment. The only notable exception is cyclohexane, where the deviation from the experimental $\Delta G_{\mathrm{hyd}}$ increases from ca. half to one kcal/mol. On the other hand, the small differences for methanol and ethanol are a bit surprising.

The increased accuracy of the Drude force field comes at a price though. First, the average CPU times for the aqueous phase simulations increase by at least a factor of two due to the additional Drude and lone pair particles. Second, additional λ points were required to achieve approximately the same level of precision as the fixed charge force field. This aspect is more thoroughly discussed in Appendix A based on the $\Delta G_{\mathrm{char}}$ calculations. The largest differences between the fixed charge and the Drude force field are found for acetamide (2.5 kcal/mol), phenol (2.35 kcal/mol), aniline (1.08 kcal/mol), benzene (1.07 kcal/mol) and water (−1.14 kcal/mol).

The $\Delta G_{\mathrm{hyd}}$ values obtained from different QM/MM methods based on trajectories with the CHARMM fixed charge force field are presented in Table 2. The columns are ordered based on the RMSD of the corresponding method from experimental hydration free energies, starting with the lowest RMSD on the left. The last six rows again represent the root mean squared deviation from experiment, the mean signed deviation and the Pearson correlation coefficient. RMSD, MSD and R${}^{2}$ are given twice: once for the complete dataset (unmarked) and once for all molecules except ethanol and acetamide (marked with asterisks). The two molecules were omitted because of the high standard deviations of more than one kcal/mol in some calculations (ethanol in the case of the fixed charge force field and acetamide because of problems encountered with the Drude force field). This allows a direct comparison of the converged parts of the two datasets.

Overall, the QM/MM results with electrostatic embedding and CHARMM TIP3P water in the MM region are slightly disappointing. The RMSD vary between 1.3 and 2.4 kcal/mol, which is worse than the pure MM result of 0.9 kcal/mol. This finding can partly be explained by the high level of optimization of the MM force field. Furthermore, the QM methods were not adapted to cancel some of the shortcomings of the TIP3P water model.

Before discussing the results in more detail, we want to validate our protocol for obtaining QM/MM hydration free energies based on the existing literature. The $\Delta G_{\mathrm{hyd}}$ values for water are in good agreement with relative free energy results between MM and QM based on QM/MM sampling with Monte Carlo simulations by Shaw, Woods and Mulholland [181]. Table 1 of [181] lists a free energy difference between QM water and CHARMM TIP3P water of 1.5±0.5 kcal/mol for MP2, while we obtain a difference of 1.9±0.2 kcal/mol. The discrepancies for BLYP (0.5±0.3 versus our 1.2±0.2 kcal/mol) and HF (2.7±0.5 versus 3.1±0.2 kcal/mol) are higher, but this can be explained by the use of different basis sets (Shaw et al. used aug-cc-pVDZ, while we employed 6-31G(d)). Furthermore, the BLYP and M06-2X $\Delta G_{\mathrm{hyd}}$ values exhibit an average deviation of 0.5 and 0.3 kcal/mol from the results published in Table 4 of [141]. The small discrepancies can be explained by the use of rigid gas-phase geometries for the solutes in [141] and by the high uncertainty of the ethanol result here. For B3LYP, the $\Delta G_{\mathrm{hyd}}$ values for ethane (1.9 kcal/mol) and methanol (−5.1 kcal/mol) are in excellent agreement with previously published hydration free energy differences (−7.0 kcal/mol here compared to −6.96 kcal/mol in Table 1 of [80] and −7.15 kcal/mol in Figure 7 of [177]). The relatively good agreement with previously published results, in conjunction with the simplicity of the solutes and the high number of QM/MM potential energy evaluations, supports our findings.

In terms of the compatibility of different QM methods with CHARMM TIP3P water based on the RMSD from experiment, the OM2 method seems to be the best (RMSD = 1.3 kcal/mol), followed by BLYP (1.4 kcal/mol), B3LYP (1.5 kcal/mol), M06-2X (1.9 kcal/mol), MP2 (2.1 kcal/mol), AM1 (2.4 kcal/mol) and HF (2.4 kcal/mol). This finding agrees with the ranking by Shaw et al. based on the free energy difference between QM and MM water (BLYP < MP2 < HF) [181]. To some degree, it is surprising that the semi-empirical method OM2 and the pure functional BLYP clearly outperform more advanced QM methods. As discussed in Section IV E and Table S14 of [141], the QM/MM electrostatics become more attractive as the amount of Hartree–Fock exchange increases from BLYP to B3LYP to M06-2X to HF/MP2. With fixed QM/MM van der Waals interactions, the hydration free energies become more negative. The MSD are −0.8 kcal/mol for BLYP, −1.0 kcal/mol for B3LYP, −1.5 kcal/mol for M06-2X and −1.9 kcal/mol for Hartree–Fock. Thus, the QM/MM results are significantly too hydrophilic. Although the CHARMM charges are based on Hartree–Fock calculations [145], the results imply that Hartree–Fock itself is not particularly suited for QM/MM simulations, due to the large systematic bias in favor of solute–solvent interactions. However, since the QM/MM $\Delta G_{\mathrm{hyd}}$ values are highly correlated with the experimental data, it is possible to address this shortcoming by scaling the interactions. This is illustrated in Appendix C.

The $\Delta G_{\mathrm{hyd}}$ values obtained from different QM/MM methods based on trajectories with the CHARMM Drude force field are presented in Table 3. The columns are again ordered based on the RMSD of the corresponding method from experimental $\Delta G_{\mathrm{hyd}}$ values, starting with the lowest RMSD on the left. Except for the two semi-empirical methods, the rank order of the QM methods based on RMSD is actually inverted, with Hartree–Fock (RMSD = 2.4 kcal/mol) followed by MP2 (2.4 kcal/mol), M06-2X (2.6 kcal/mol), B3LYP (2.7 kcal/mol) and BLYP (3.1 kcal/mol). However, the RMSD is not a reliable measure here, since the acetamide results are far from converged, with standard deviations between 1.4 and 2.9 kcal/mol. As more thoroughly discussed in a recent paper, high standard deviations in multi-scale free energy simulations can be an indicator that the MM energy minimum is very far away from the QM energy minimum [133]. Indeed, when comparing the optimal C–C bond length of acetamide of MM (1.153 Å for the types CD201A and CD33C) with, e.g., the bond length of an energy optimized structure with OM2 (1.513 Å), there is a clear discrepancy of 0.36 Å, which leads to substantial energy differences. Given that the equilibrium bond lengths of C–C bonds are typically between 1.35 and 1.55 Å in the CHARMM force field, this is a clear indication for a typo in the Drude parameter file for acetamide. A further investigation is in progress.

Ignoring the flawed acetamide results and focusing on the metrics marked with an asterisk, the overall results of most methods (except BLYP and AM1) are surprisingly similar, with RMSD * between 1.3 and 1.5 kcal/mol and MSD * between mere −0.4 and 0.5 kcal/mol. While the RMSD * are a little bit higher than the best results for the CHARMM TIP3P water model (RMSD * between 0.9 and 2.4 kcal/mol), the consistency between most methods and the low systematic errors can be regarded as a sign of better compatibility with QM/MM methods. Given that the development of polarizable Drude force fields is still in its early stages, one can still expect some improvements in the future. The AM1 semi-empirical method is among the most inaccurate methods in the test set, with RMSD of 2.4 kcal/mol for the fixed charge model and 3.1 kcal/mol for the Drude model. In the light of such results, it is somewhat surprising that the popular AM1-BCC method to determine MM charges [182,183], which builds upon AM1, is as effective as it is when it comes to hydration free energies [44].

Another aspect that can influence the accuracy of the Drude oscillator model is the use of the extended Lagrangian formalism [184], in which Drude particles have a mass and kinetic energy. This implies that the particles do not necessarily reside at the energy minimum at each step. Also in our QM/MM energy evaluations, the Drude particles in the MM region were not relaxed in response to the QM wave function. To evaluate the effect of relaxing the Drude particles, five steps of conjugate gradient energy minimization were performed with QM/MM after an MM SCF optimization of the Drude particles. The resulting hydration free energies for Hartree–Fock with the extended Lagrangian approach (HF-EL) and based on the self-consistent optimization of the Drude particles (HF-SCOD) are shown in Table 4. While the overall agreement with experiment in terms of the RMSD does not change significantly with the use of self-consistent Drude particles (RMSD of 2.4 and 2.5 kcal/mol), the solvent affinity increases in all cases (as it should). For the Hartree–Fock calculations, this leads to a lower systematic error in terms of MSD of a mere 0.04 kcal/mol (instead of 0.34 kcal/mol). The average change of 0.3 kcal/mol is lower than the average standard deviation of ca. 0.7 kcal/mol, so most differences here are not statistically significant.

**Figure 2:**
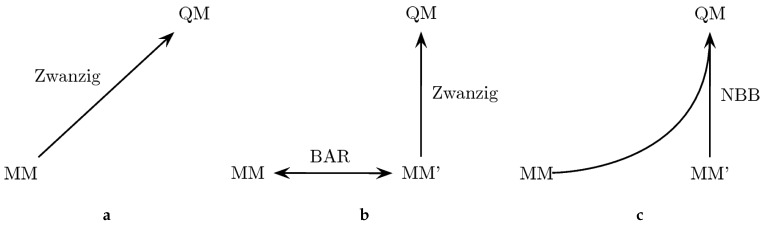
Three ways to calculate the free energy difference between an MM state and a QM state: (**a**) calculating the free energy difference between an MM end state and QM with the Zwanzig equation; (**b**) generating a tailored force field (MM’) with better overlap with the QM target to calculate the free energy difference; (**c**) combining data from both MM, MM’ and QM.

Because the convergence of some of the QM/MM $\Delta G_{\mathrm{hyd}}$ results was not satisfactory, we also explored the possibility to improve this situation by employing a tailored force field (denoted as MM’). By adopting bonded terms that match more closely the bond lengths and angles encountered in the target QM method, the phase space overlap is supposed to be increased, which also improves the convergence of the free energy calculation between MM and QM [133]. The approach is outlined in Figure 2. In particular, we explored three different ways to perform the “bookend” corrections: (a) using the Zwanzig equation [178] to directly calculate the free energy difference between the MM force field and the QM Hamiltonian; (b) generating an MM’ tailored force field with optimized parameters to increase the phase space overlap with the QM Hamiltonian; the free energy difference between the original force field and the tailored force field can be calculated with Bennett’s acceptance ratio method (BAR) [161], while the free energy difference between the modified MM’ force field and the QM state is calculated with the Zwanzig equation; (c) combining all the potential energy data from MM, MM’ and QM with the Non-Boltzmann–Bennett equation [79,80,180].

A comparison of the results of the three theoretically equivalent approaches is given in Table 5. The third column (MM→QM) reflects the $\Delta G_{\mathrm{hyd}}$ values from direct free energy calculations between MM and QM energy surfaces using the Zwanzig equation. In principle, the results here should correspond to those in the third column of Table 2 (OM2). However, since different trajectories and setups were employed, one can expect some small discrepancies. The overall RMSD (1.5 compared to 1.3 kcal/mol) and MSD (0.3 versus −0.3 kcal/mol) are similar compared to Table 2, which serves as another verification of the approach. The third column of Table 5 shows the results obtained using the tailored MM’ force field to calculate the free energy difference to the QM state. The $\Delta G_{\mathrm{hyd}}$ values of Columns 2 and 3 should also match within the corresponding uncertainties, since the end points are the same. Indeed, except for aniline, the differences between the two columns are below 0.2–0.3 kcal/mol, which also corresponds to the average standard deviation of the results (shown in the last line). Importantly, the average standard deviation is a little bit lower for the MM→MM’→QM transformation, due to the increased phase space overlap between the MM’ and the QM state. The last column shows the result of an NBB calculation that combines the potential energy data of the two transformations in an optimal way. The fact that the NBB results are almost identical to the MM→MM’→QM transformation further indicates that there is more phase space overlap between the MM’ and QM state, thus dominating the NBB calculation. However, the overall improvement is rather small, which signifies that the original bonded parameters were already well optimized.

## 4. Conclusions

In this work, we computed hydration free energies for twelve simple solutes to determine an effective choice of QM method to use in combination with explicit solvent. Here, we focused on the fixed charge CHARMM TIP3P and the polarizable SWM4 water model in the CHARMM force field. As a reference, we first provided hydration free energies based on pure MM simulations. Both the fixed charge (RMSD = 0.89 kcal/mol) and the Drude force field simulations (RMSD = 0.55 kcal/mol) exhibit excellent agreement with the experimental data and are well converged with respect to conformational sampling.

For QM/MM hydration free energy calculations based on the CHARMM CGenFF fixed charge force field, the best results were obtained with the OM2 semi-empirical method (RMSD = 1.3 kcal/mol) and the BLYP method (RMSD = 1.4 kcal/mol). The other methods (B3LYP, M06-2X, MP2, AM1 and Hartree–Fock) yielded RMSD between 1.5 and 2.4 kcal/mol. This ranking of QM methods agrees with the previous observation that the systematic error of hydration free energies of QM/MM methods with CHARMM TIP3P water increases systematically with the amount of Hartree–Fock exchange [141]. Therefore, we recommend using either OM2 or BLYP for QM/MM simulations in aqueous solution with CHARMM TIP3P water. This QM/MM protocol was also successfully applied to the calculation of distribution coefficients in SAMPL5 [130], which reflects the change from a hydrophilic to a hydrophobic environment.

As for the QM/MM hydration free energy calculations based on the CHARMM Drude force field, the best results were obtained with the OM2 semi-empirical method (RMSD = 1.6 kcal/mol). However, the ranking of the other methods is nearly reversed, with Hartree–Fock (RMSD = 2.4 kcal/mol) outperforming MP2, M06-2X, B3LYP, BLYP and AM1. The MP2, M06-2X and Hartree–Fock methods perform slightly better with the Drude force field in terms of RMSD, and their systematic error is significantly lower. Thus, if a potential bias from the solute–solvent interactions is a concern, it might be advisable to employ the Drude force field for QM/MM simulations with those methods. However, the performance of QM/MM with the Drude force field is only marginally better. Furthermore, the Drude accuracy between the extended-Lagrangian (EL) and self-consistent optimization implementations is statistically indistinguishable, but can slightly affect the systematic bias.

Overall, the OM2 semi-empirical method shows the best performance for both datasets with RMSD of 1.3 and 1.6 kcal/mol, while the AM1 semi-empirical method exhibits the worst performance with RMSD of 2.4 and 3.1 kcal/mol. The PM3 semi-empirical method was omitted in the manuscript because of its RMSD of 3.5 and 4.5 kcal/mol, further demonstrating the high variability in the quality of semi-empirical methods. However, both the accuracy and robustness of the OM2 hydration free energy results are very encouraging, especially since the OM2 parametrization did not include solvation free energies. This makes the method suitable for improving the quality of MM free energy predictions via post-processing, as OM2 can be applied to thousands of snapshots within mere minutes on a commodity laptop.

Our results also corroborate the conclusions of a recent study by Ganguly, Boulanger and Thiel [185]. The effect of MM polarization via Drude particles on QM/MM hydration free energies is only moderate compared to the well-developed CHARMM fixed charge force field. Fixed charge force fields are well tested, faster and more robust than the recently developed polarizable force fields. Therefore, they will most likely continue to play a significant role in computational chemistry. While polarization is a highly relevant physical effect, Drude force fields still neglect other important factors such as charge penetration, coupling of polarization with many-body exchange, dispersion and charge transfer [186,187,188]. In addition, the impact of Drude point charges in proximity to the QM region is still unclear at this point.

The force field parameters (e.g., the van der Waals parameters) will likely have to be adapted according to the target QM function. Thus, some form of tailored MM’ force field will be beneficial for future applications of QM/MM in multi-scale free energy simulations. The need for improvement is highlighted by the systematic errors of QM/MM in the kcal/mol range, as well as the clear superiority of the MM $\Delta G_{\mathrm{hyd}}$ results compared to QM/MM. Our results show that spending computer power to add all the right physics to the QM region in a QM/MM simulation will be in vain if the MM description of the solvent environment is not compatible with the QM description of the solute.

## Figures and Tables

**Table 1:** Hydration free energies obtained with the CHARMM fixed charge and the CHARMM Drude force field in kcal/mol.

Molecule	Expt. ^a^	$\Delta G_{\mathrm{hyd}}^{\mathrm{FC}}$ ^b^	$\Delta G_{\mathrm{hyd}}^{\mathrm{Drude}}$ ^c^	$\Delta \Delta G_{\mathrm{FC}-\mathrm{Drude}}$ ^d^
water	−6.31	−6.91 ± 0.04	−5.77 ± 0.02	−1.14
methanol	−5.10	−4.68 ± 0.02	−4.90 ± 0.03	0.21
ethanol	−5.05	−4.62 ± 0.08	−4.65 ± 0.05	0.02
methanethiol	−1.24	−0.23 ± 0.01	−1.04 ± 0.03	0.80
acetamide	−9.68	−8.15 ± 0.06	−10.64 ± 0.06	2.50
tetrahydrofuran	−3.47	−2.55 ± 0.05	−3.12 ± 0.03	0.57
benzene	−0.86	−0.29 ± 0.03	−1.36 ± 0.05	1.07
phenol	−6.61	−4.72 ± 0.07	−7.07 ± 0.03	2.35
aniline	−5.49	−5.05 ± 0.04	−6.13 ± 0.05	1.08
ethane	1.83	2.23 ± 0.01	2.16 ± 0.02	0.07
hexane	2.48	2.77 ± 0.07	2.54 ± 0.08	0.23
cyclohexane	1.23	1.77 ± 0.04	2.15 ± 0.04	−0.38
RMSD ^e^		0.89	0.53	
MSD ^f^		0.65	0.04	
R${}^{2}$ ^g^		0.97	0.99	

^a^ Experimental hydration free energies. ^b^ Hydration free energies obtained with the CHARMM fixed charge force field. ^c^ Hydration free energies obtained with the CHARMM Drude force field. ^d^ Difference between fixed charge and the Drude model. ^e^ Root mean squared deviation from experimental data. ^f^ Mean signed deviation from experimental data. ^g^ Square of the Pearson correlation coefficient between calculated and experimental hydration free energies.

**Table 2:** Hydration free energies of QM/MM with different QM methods based on trajectories of the CHARMM fixed charge force field.

Molecule	Expt. ^a^	OM2	BLYP	B3LYP	M06-2X	MP2	AM1	HF
water	−6.31	−4.4±0.2	−8.1±0.2	−8.8±0.2	−9.6±0.2	−8.8±0.2	−2.3±0.2	−10.0±0.2
methanol	−5.10	−4.2±0.4	−5.1±0.2	−5.1±0.1	−5.5±0.1	−5.8±0.1	−1.6±0.1	−6.3±0.2
ethanol	−5.05	−6.7±1.3	−8.5±1.8	−7.0±1.8	−7.2±1.8	−8.1±2.0	−2.0±0.4	−6.6±0.8
methanethiol	−1.24	−0.8±0.2	−2.6±0.4	−3.0±0.3	−3.2±0.2	−3.2±0.3	−4.0±0.2	−3.5±0.1
acetamide	−9.68	−12.7±0.6	−11.4±0.9	−12.2±0.6	−12.9±0.6	−13.8±0.5	−8.4±0.4	−14.8±0.7
tetrahydrofuran	−3.47	−4.5±0.6	−3.4±0.3	−4.2±0.2	−4.6±0.3	−5.3±0.3	−2.2±0.5	−5.9±0.4
benzene	−0.86	−2.1±0.2	−0.5±0.2	−0.9±0.2	−1.4±0.2	−1.5±0.2	0.1±0.3	−1.8±0.2
phenol	−6.61	−5.5±0.5	−6.0±0.4	−6.9±0.3	−7.7±0.3	−7.7±0.4	−2.7±0.3	−8.3±0.5
aniline	−5.49	−6.0±0.4	−7.3±0.8	−7.9±0.4	−8.7±0.3	−9.0±0.4	−3.6±0.6	−8.8±0.3
ethane	1.83	1.8±0.1	2.0±0.1	1.9±0.1	1.9±0.1	1.7±0.1	2.1±0.1	1.9±0.1
*n*-hexane	2.48	1.8±0.4	2.3±0.3	2.1±0.3	1.9±0.3	1.5±0.3	2.5±0.4	2.1±0.4
cyclohexane	1.23	0.9±0.5	1.2±0.2	1.1±0.2	1.1±0.2	0.8±0.2	1.3±0.3	1.0±0.5
RMSD ^b^		1.3	1.4	1.5	1.9	2.1	2.4	2.4
MSD ^c^		−0.3	−0.8	−1.0	−1.5	−1.7	1.5	−1.9
R${}^{2}$ ^d^		0.90	0.94	0.97	0.97	0.97	0.74	0.97
RMSD *		1.0	0.9	1.3	1.7	1.7	2.4	2.0
MSD *		0.1	−0.4	−0.8	−1.2	−1.4	1.3	−1.6
R${}^{2}$ *		0.92	0.95	0.95	0.95	0.96	0.65	0.97

^a^ Experimental hydration free energies. ^b^ Root mean squared deviation from experimental data. ^c^ Mean signed deviation from experimental data. ^d^ Square of the Pearson correlation coefficient between calculated and experimental hydration free energies. * marks results that exclude ethanol and acetamide due to the associated high uncertainties.

**Table 3:** Hydration free energies of QM/MM with different QM methods based on trajectories of the CHARMM Drude force field.

Molecule	Expt. ^a^	OM2	HF	MP2	M06-2X	B3LYP	BLYP	AM1
water	−6.31	−2.8±0.2	−6.3±0.2	−4.4±0.2	−5.4±0.2	−4.6±0.2	−3.2±0.2	−1.8±0.2
methanol	−5.10	−3.2±0.2	−4.7±0.3	−4.6±0.4	−4.3±0.2	−3.7±0.2	−3.3±0.3	−1.7±0.2
ethanol	−5.05	−2.9±0.4	−3.5±0.4	−3.6±0.2	−3.2±0.3	−2.7±0.2	−2.3±0.4	−0.7±0.5
methanethiol	−1.24	−1.0±0.2	−2.8±0.4	−1.8±0.2	−2.0±0.3	−1.4±0.3	−0.1±0.4	−2.0±0.2
acetamide	−9.68	−7.5±2.7	−2.6±2.2	−2.8±1.3	−1.9±1.6	−1.5±1.6	−0.6±1.7	−3.0±2.9
tetrahydrofuran	−3.47	−3.8±0.5	−2.6±0.3	−2.3±0.2	−1.6±0.2	−1.2±0.2	−0.5±0.3	−1.6±0.5
benzene	−0.86	−2.3±0.3	−1.1±0.4	−0.9±0.2	−0.7±0.3	−0.4±0.2	0.0±0.2	−0.3±0.3
phenol	−6.61	−8.1±0.8	−9.9±1.2	−10.4±0.4	−9.5±0.9	−8.8±0.7	−8.4±0.4	−3.8±0.4
aniline	−5.49	−5.8±0.6	−7.4±1.1	−7.1±0.4	−7.1±0.7	−6.1±0.5	−5.5±0.5	−3.2±0.5
ethane	1.83	1.9±0.1	2.2±0.2	2.4±0.2	2.3±0.2	2.4±0.2	2.9±0.2	2.5±0.2
hexane	2.48	2.4±0.3	2.5±0.6	2.7±0.3	2.9±0.3	2.9±0.3	3.3±0.3	2.7±0.3
cyclohexane	1.23	1.8±0.3	1.9±0.3	2.0±0.2	2.1±0.2	2.2±0.2	2.5±0.2	1.9±0.3
RMSD ^b^		1.6	2.4	2.5	2.6	2.7	3.2	3.2
MSD ^c^		0.6	0.3	0.6	0.8	1.3	1.9	2.3
R${}^{2}$ ^d^		0.85	0.63	0.64	0.61	0.61	0.57	0.78
RMSD *		1.4	1.4	1.5	1.3	1.3	1.7	2.2
MSD *		0.3	−0.5	−0.1	0.0	0.5	1.1	1.6
R${}^{2}$ *		0.82	0.92	0.88	0.90	0.90	0.87	0.84

^a^ Experimental hydration free energies. ^b^ Root mean squared deviation from experimental data. ^c^ Mean signed deviation from experimental data. ^d^ Square of the Pearson correlation coefficient between calculated and experimental hydration free energies. * marks results that exclude ethanol and acetamide due to the associated high uncertainties.

**Table 4:** Comparison of the QM/MM hydration free energies with Hartree–Fock based on the extended Lagrangian formalism (HF-EL) and with a self-consistent optimization of the Drude particles (HF-SCOD) in kcal/mol.

Molecule	Expt. ^a^	HF-EL ^b^	HF-SCOD ^c^	Diff ^d^
water	−6.31	−6.3±0.1	−6.5±0.1	0.2
methanol	−5.10	−4.7±0.4	−4.8±0.3	0.1
ethanol	−5.05	−3.5±0.5	−3.7±0.5	0.2
methanethiol	−1.24	−2.8±0.4	−3.1±0.4	0.3
acetamide	−9.68	−2.8±2.2	−2.7±2.2	0.0
tetrahydrofuran	−3.47	−2.6±0.3	−2.8±0.3	0.3
benzene	−0.86	−1.1±0.7	−1.6±0.7	0.5
phenol	−6.61	−9.9±1.2	−10.2±1.0	0.3
aniline	−5.49	−7.2±1.2	−7.8±1.2	0.6
ethane	1.83	2.2±0.2	1.9±0.2	0.3
*n*-hexane	2.48	2.6±0.6	2.1±0.6	0.5
cyclohexane	1.23	1.9±0.3	1.5±0.3	0.4
RMSD ^e^		2.4	2.5	
MSD ^f^		0.3	0.0	

^a^ Experimental hydration free energies. ^b^ QM/MM hydration free energies with Hartree–Fock/6-31G(d) based on CHARMM Drude trajectories using the extended Lagrangian formalism. ^c^ QM/MM hydration free energies with Hartree–Fock/6-31G(d) based on CHARMM Drude trajectories after self-consistent optimization in the post-processing step. ^d^ Difference between the extended Lagrangian and the self-consistent Drude particle results. ^e^ Root mean squared deviation from experimental data. ^f^ Mean signed deviation from experimental data.

**Table 5:** Comparison of the three different approaches to obtain QM/MM hydration free energies with OM2 and the fixed charge force field.

Molecule	Expt. ^a^	MM→QM ^b^	MM→MM’→QM ^c^	NBB ^d^
water	−6.31	−4.4±0.2	−4.5±0.2	−4.4±0.2
methanol	−5.10	−2.7±0.1	−2.9±0.1	−2.8±0.1
ethanol	−5.05	−3.0±0.3	−3.1±0.3	−3.1±0.3
methanethiol	−1.24	−0.7±0.1	−0.7±0.1	−0.7±0.1
acetamide	−9.68	−12.7±0.4	−12.5±0.4	−12.5±0.4
tetrahydrofuran	−3.47	−3.9±0.5	−4.1±0.4	−4.2±0.4
benzene	−0.86	−2.0±0.2	−2.1±0.2	−2.0±0.2
phenol	−6.61	−5.0±0.5	−5.2±0.3	−5.2±0.3
aniline	−5.49	−5.1±0.5	−5.8±0.2	−5.8±0.2
ethane	1.83	1.8±0.1	1.8±0.1	1.8±0.1
hexane	2.48	1.7±0.3	1.6±0.4	1.6±0.4
cyclohexane	1.23	1.0±0.2	0.9±0.2	0.9±0.2
RMSD ^e^		1.5	1.5	1.5
MSD ^f^		0.3	0.2	0.2
$\langle SD\rangle$ ^g^		0.3	0.2	0.2

^a^ Experimental hydration free energies. ^b^ QM/MM hydration free energies obtained with the Zwanzig equation based on the CHARMM fixed charge force field. ^c^ QM/MM hydration free energies obtained with the Zwanzig equation based on a tailored force field that matches the gas phase bond lengths and angles, plus the correction for the free energy change between the original force field and the tailored force field. ^d^ QM/MM hydration free energies obtained with the NBB equation based on data from both the original force field and the tailored force field. ^e^ Root mean squared deviation from experimental data. ^f^ Mean signed deviation from experimental data. ^g^ Average standard deviation of simulations.

**Figure A1:**
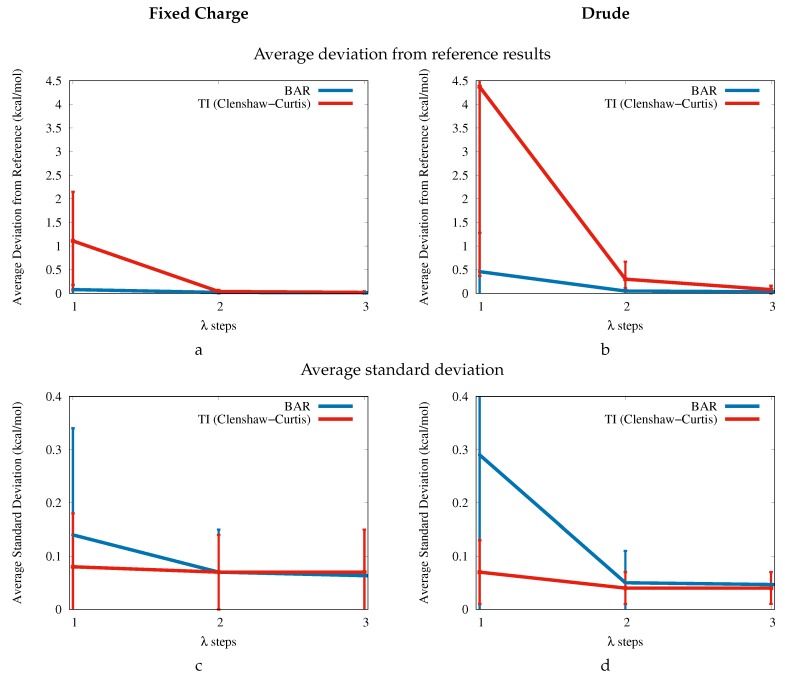
Convergence of the free energy of uncharging ($\Delta G_{\mathrm{char}}$) with respect to the number of λ steps. (**a**) Average deviation from reference results for all twelve molecules using the fixed charge force field. (**b**) Average deviation from reference results using the Drude force field. (**c**) Average standard deviation using the fixed charge force field. (**d**) Average standard deviation using the Drude force field.

## Abbreviations

The following abbreviations are used in this manuscript:

$\Delta G_{\mathrm{hyd}}$	Hydration free energy
$\Delta G_{\mathrm{char}}$	Free energy difference of removing the charges of the solute
$\Delta G_{\mathrm{vdw}}$	Free energy difference of removing the Lennard–Jones interactions of the uncharged solute
BAR	Bennett’s acceptance ratio
CGenFF	CHARMM General Force Field
EL	Extended Lagrangian
HF	Hartree–Fock
MM	Molecular mechanics
MP2	second-order Møller–Plesset
MSD	Mean-signed deviation
NBB	Non-Boltzmann–Bennett
PBC	Periodic boundary conditions
QM	Quantum mechanics
R${}^{2}$	Pearson’s correlation coefficient squared
RMSD	Root mean squared deviation
SCF	Self-consistent field
SCOD	Self-consistent optimized Drude
SD	Standard deviation
SQM	Semi-empirical QM
TI	Thermodynamic integration

## Appendix A. Convergence of MM Charge Change Simulations

Figure A1 shows a comparison of the convergence of the $\Delta G_{\mathrm{char}}$ free energy estimates of the fixed charge (left side) and Drude force field (right). The first row shows the average deviation of all twelve molecules with respect to a reference BAR protocol with four λ steps (λ = 0.00, 0.25, 0.50, 0.75, 1.00). Each λ point uses 1900 snapshots from 1.9 ns of simulation time, and each simulation was repeated four times to obtain standard deviations. The second row shows the average standard deviation of the free energy estimates. In addition, each plot also shows a comparison of BAR free energy estimates (blue) with the corresponding estimates based on Thermodynamic Integration (TI) [189] using Clenshaw–Curtis numerical quadrature (red) [190]. Clenshaw–Curtis quadrature is an attractive option for Thermodynamic Integration because it uses the physical end points of the simulation and is nestable. In addition, it is almost as efficient as the well-known Gauss–Legendre method [191,192,193]. In contrast to Gauss–Legendre quadrature, which does not include the physical end points, Clenshaw–Curtis quadrature allows a direct comparison to BAR, using exactly the same potential energy data.

Focusing on the average deviation of the free energy estimates (top of Figure A1), one can observe that more λ steps are necessary with the Drude force field in order to yield the same level of accuracy and precision as the fixed charge force field. The TI results based on one λ step only use the physical end points, which implicitly assumes that the $\frac{\partial G}{\partial \lambda }$ is linear. Thus, the errors of the one-step TI protocol are an indicator for higher-order coupling within the system and that the probability distribution of the potential energy difference cannot be described by a Gaussian [30,194]. Not surprisingly, this problem is more pronounced in the polarizable Drude force field with an average error of 4.37 kcal/mol of the one-step protocol (compared to an error of 1.11 kcal/mol for the fixed charge force field). This suggests that linear response methods such as the Linear Interaction Energy method [195] or low-order cumulant expansion [196] are only marginally compatible with the Drude force field.

The average standard deviation (bottom of Figure A1) is elevated for the one-step BAR protocol (0.14 kcal/mol for the fixed charge force field and 0.29 kcal/mol for the Drude force field), while all other protocols exhibit average standard deviations between 0.04 and 0.08 kcal/mol. This demonstrates that the precision is not a reliable measure for the accuracy of the free energy estimates. This is especially striking for TI, where the main source of error arises from the treatment of the higher derivatives of the integrand. This notwithstanding, both BAR and TI can reach converged values of $\Delta G_{\mathrm{char}}$ with just two or three λ steps for the molecules considered here. Our choice of using more λ points than necessary was motivated by the exchange rate of the Hamiltonian replica exchange scheme to increase sampling.

## Appendix B. Detailed MM Free Energy Results

**Table A1:** Free energy differences of all substeps of the hydration free energy calculations with the CHARMM fixed charge force field in kcal/mol.

Molecule	$\Delta G_{\mathrm{gas}}$ ^a^	$\Delta G_{\mathrm{char}}$ ^b^	Ovl ^c^	$\Delta G_{\mathrm{vdw}}$ ^d^	Ovl ^c^	$\Delta G_{\mathrm{hyd}}$ ^e^
water	0.000 ± 0.000	8.99 ± 0.04	33	−2.08 ± 0.01	77	−6.91 ± 0.04
methanol	−15.284 ± 0.001	−8.84 ± 0.02	44	−1.76 ± 0.01	64	−4.68 ± 0.02
ethanol	5.217 ± 0.004	11.76 ± 0.02	43	−1.92 ± 0.08	54	−4.62 ± 0.08
methanethiol	−5.060 ± 0.001	−3.94 ± 0.01	82	−0.88 ± 0.01	61	−0.23 ± 0.01
acetamide	78.514 ± 0.004	88.01 ± 0.03	31	−1.35 ± 0.05	53	−8.15 ± 0.06
tetrahydrofuran	0.400 ± 0.001	4.12 ± 0.01	56	−1.17 ± 0.05	44	−2.55 ± 0.05
benzene	−13.300 ± 0.004	0.00 ± 0.01	76	−13.02 ± 0.03	37	−0.29 ± 0.03
phenol	−0.592 ± 0.003	16.79 ± 0.01	48	−12.66 ± 0.07	33	−4.72 ± 0.07
aniline	8.049 ± 0.004	25.89 ± 0.01	47	−12.79 ± 0.04	31	−5.05 ± 0.04
ethane	−8.861 ± 0.001	−8.94 ± 0.00	98	−2.15 ± 0.01	59	2.23 ± 0.01
*n*-hexane	−5.165 ± 0.005	−4.69 ± 0.01	94	−3.24 ± 0.06	31	2.77 ± 0.07
cyclohexane	−1.584 ± 0.002	−2.29 ± 0.00	96	−1.06 ± 0.04	32	1.77 ± 0.04

^a^ Free energy difference associated with turning off all non-bonded interactions in the gas phase. ^b^ Free energy difference of uncharging the solute in aqueous solution. ^c^ Smallest BAR overlap integral of all λ-steps in % (cf. [133,161,197,198]). ^d^ Free energy difference of removing all Lennard–Jones interactions of the uncharged solute in aqueous solution. ^e^ Total hydration free energy (cf. Table 1).

**Table A2:** Free energy differences of all substeps of the hydration free energy calculations with the CHARMM Drude force field in kcal/mol.

Molecule	$\Delta G_{\mathrm{gas}}$ ^a^	$\Delta G_{\mathrm{char}}$ ^b^	Ovl ^c^	$\Delta G_{\mathrm{vdw}}$ ^d^	Ovl ^c^	$\Delta G_{\mathrm{hyd}}$ ^e^
water	0.000 ± 0.000	7.92 ± 0.02	52	−2.15 ± 0.02	78	−5.77 ± 0.02
methanol	−11.646 ± 0.003	−5.33 ± 0.01	57	−1.42 ± 0.03	51	−4.90 ± 0.03
ethanol	−0.931 ± 0.003	6.08 ± 0.01	51	−2.36 ± 0.05	35	−4.65 ± 0.05
methanethiol	−2.528 ± 0.000	−0.28 ± 0.01	83	−1.21 ± 0.03	32	−1.04 ± 0.03
acetamide	104.139 ± 0.004	119.42 ± 0.01	29	−4.63 ± 0.05	35	−10.64 ± 0.06
tetrahydrofuran	0.648 ± 0.002	4.61 ± 0.02	63	−0.84 ± 0.03	20	−3.12 ± 0.04
benzene	−22.578 ± 0.003	0.98 ± 0.01	76	−22.20 ± 0.05	8	−1.36 ± 0.05
phenol	−16.280 ± 0.010	11.82 ± 0.02	44	−21.03 ± 0.02	7	−7.07 ± 0.03
aniline	−7.691 ± 0.014	19.98 ± 0.02	43	−21.54 ± 0.05	7	−6.13 ± 0.06
ethane	−3.769 ± 0.001	−3.64 ± 0.00	95	−2.29 ± 0.02	43	2.16 ± 0.02
*n*-hexane	−3.597 ± 0.005	−2.39 ± 0.01	79	−3.75 ± 0.08	7	2.54 ± 0.08
cyclohexane	−0.026 ± 0.002	−0.09 ± 0.00	84	−2.09 ± 0.04	7	2.15 ± 0.04

^a^ Free energy difference associated with turning off all non-bonded interactions in the gas phase. ^b^ Free energy difference of uncharging the solute in aqueous solution. ^c^ Smallest BAR overlap integral of all λ-steps in % (cf. [133,161,197,198]). ^d^ Free energy difference of removing all Lennard–Jones interactions of the uncharged solute in aqueous solution. ^e^ Total hydration free energy (cf. Table 1).

## Appendix C. Scaling the Hydration Free Energies

The high correlation coefficient between the QM/MM derived hydration free energies and the experimental data in Table 2 indicates that a better agreement with experiment can be achieved by scaling the results. Using the ratio of the experimental chemical potential of water in its own liquid relative to the computational result, i.e., $\frac{\Delta G_{hyd}^{water\;Expt}}{\Delta G_{hyd}^{water}}$, as a scaling factor, one can obtain significantly better agreement with experiment. Table A3 illustrates this for the QM/MM results with the fixed charge force field and Hartree–Fock (which yielded the worst RMSD with 2.4 kcal/mol and an MSD of −1.9 kcal/mol). The employed scaling factor was 0.6317. Interestingly, the RMSD drops to 0.8 kcal/mol and the MSD to a mere −0.02 kcal/mol. This is an indicator that the scaling factor might be transferable. The results also show that the bias of QM/MM $\Delta G_{\mathrm{hyd}}$ values is not constant and, therefore, does not cancel if the end points of a free energy calculation involve different chemical species (such as in chemical reactions).

**Table A3:** Hartree–Fock QM/MM hydration free energies scaled by $\frac{\Delta G_{hyd}^{water\;Expt}}{\Delta G_{hyd}^{water\;HF}}=0.6317$. All results are in kcal/mol.

Molecule	Expt.	$\Delta G_{\mathrm{hyd}}$
water	−6.31	−6.31±0.20
methanol	−5.10	−4.01±0.16
ethanol	−5.05	−4.15±0.81
methanethiol	−1.24	−2.19±0.15
acetamide	−9.68	−9.38±0.66
tetrahydrofuran	−3.47	−3.71±0.39
benzene	−0.86	−1.11±0.20
phenol	−6.61	−5.22±0.51
aniline	−5.49	−5.58±0.33
ethane	1.83	1.19±0.13
*n*-hexane	2.48	1.36±0.37
cyclohexane	1.23	0.62±0.52
RMSD		0.80
MSD		−0.02
R${}^{2}$		0.97
